# Prevalence, Location, and Interference With Daily Life of Chronic Pain in Long-Term Survivors After Discharge From a Tertiary Emergency Center

**DOI:** 10.7759/cureus.35382

**Published:** 2023-02-23

**Authors:** Naoya Hashimoto, Takeshi Unoki, Nozomi Nagano, Ryota Funamizu, Keigo Sawamoto

**Affiliations:** 1 Advanced Critical Care and Emergency Center, Sapporo Medical University Hospital, Sapporo, JPN; 2 Acute and Critical Care Nursing, Graduate School of Nursing, Sapporo City University, Sapporo, JPN; 3 Department of Acute and Critical Care Nursing, School of Nursing, Sapporo City University, Sapporo, JPN; 4 Doctoral Program, Graduate School of Nursing, Sapporo City University, Sapporo, JPN; 5 Department of Emergency Medicine, Sapporo Medical University, Sapporo, JPN

**Keywords:** persistent pain, emergency medicine, post-intensive care syndrome, critical illness, critical care, intensive care units, chronic pain

## Abstract

Background

This study aimed to investigate the prevalence, location, and characteristics of new-onset chronic pain by using a new definition in long-term survivors after discharge from a tertiary emergency center.

Materials and methods

We conducted a single-center ambidirectional cohort study from January to May 2022. A survey of patients was conducted by postal mail two to 2.5 years after their discharge from a tertiary emergency center. We used the Brief Pain Inventory to investigate chronic pain parameters, and the painDETECT questionnaire to investigate neuropathic pain. Patient information during hospitalization was collected retrospectively from medical records.

Results

The survey was sent to 78 patients, 63 (81%) of whom responded and were included in the analysis. Nine of the 63 patients (14%) had new-onset chronic pain. Of these, six (67%) had chronic pain of moderate or severe intensity which interfered with daily life. The most frequent location of chronic pain was the foot/ankle (n=4, 44%). Neuropathic pain was present in four (44%) patients with new-onset chronic pain.

Conclusion

New-onset chronic pain may occur for up to two to 2.5 years after discharge from a tertiary emergency center, and this may interfere with daily life. Therefore, a follow-up system for chronic pain is warranted.

## Introduction

Chronic pain is an important issue, especially for patients who have experienced critical illness. A systematic review of persistent pain after critical illness reported that 28-77% of patients experience chronic pain [1]. In addition, multiple studies have reported the location, intensity, and interference with daily life of the pain after critical illness [2-5]. Chronic pain after critical illness is an essential issue because it may be associated with mental health disorders such as anxiety and depression [6] and lower health-related quality of life (HRQOL) [7,8].

However, prior studies have had several limitations. First, each study had a different definition of chronic pain. In 2015, the International Association for the Study of Pain (IASP) updated the definition of chronic pain to "Pain that lasts or recurs for longer than 3 months" [9]. However, no studies have used this updated definition. Second, there is a lack of knowledge of long-term survivors beyond two years after ICU discharge. Most previous studies have examined survivors for only up to one year after ICU discharge. Third, most studies have been conducted in general ICU patients and rarely in emergency patients.

Therefore, this study aimed to determine the prevalence of new-onset chronic pain among long-term survivors after discharge from a tertiary emergency center using the new IASP definition. Our purpose was to determine the intensity, location, and interference with daily life of new-onset chronic pain and the presence of neuropathic pain.

This article was previously posted to the “Preprints” preprint server on December 20, 2022 (https://www.preprints.org/manuscript/202212.0358/v1).

## Materials and methods

We conducted a single-center, ambidirectional cohort study. A survey questionnaire was sent to long-term survivors, two to 2.5 years after discharge from an emergency center, to determine their chronic pain status. In addition, patient information was collected retrospectively from medical records and during hospitalization. Chronic pain was defined as "Pain that lasts or recurs for longer than 3 months" [9]. The study was conducted according to the Statement for the Improvement of the Quality of Observational Epidemiological Study Reporting (STROBE) [10].

Setting

This study was conducted at Sapporo Medical University Hospital's Advanced Critical Care and Emergency Center (Emergency Center). This facility is a tertiary emergency medical care institution to which severely ill patients requiring intensive care are transported, according to Japanese domestic standards [11]. The emergency center has six ICU beds and 12 high-dependency care unit (HCU) beds. Patients admitted to this emergency center are mainly those transported to the hospital, and only those with critical illnesses or severe trauma are admitted. After emergency transport, patients are managed in the ICU or HCU, depending on the severity of their condition. However, patients with cardiovascular surgical conditions are not admitted to this emergency center because they are managed in another medical-surgical ICU in the hospital.

Participants

We surveyed patients who were discharged from the emergency center between July 8, 2019, and February 29, 2020. The study period was from January 8, 2022, to May 5, 2022.

Exclusion criteria were as follows: (1) patients aged <18 years at the time of the survey, (2) patients with severe cognitive or consciousness impairment according to their medical records, (3) patients who were readmitted to the emergency center between discharge from the emergency center and the time of survey, (4) patients who were transferred to the emergency center after >24 hours in the ICU of another facility, (5) patients who could not understand Japanese, (6) patients who refused to have the survey mailed to them during the telephone screening, and (7) patients who were unable to answer the self-administered questionnaire (such as those with cognitive dysfunction), (8) patients who were hospitalized or institutionalized in a medical institution or nursing facility at the time of the survey (9) patients who could not be contacted by telephone (10) patients who were dead at the time of the survey (11) patients who were admitted to the medical facility for trauma, or were surgically admitted to the hospital, or admitted to the ICU, between discharge from the emergency center and the time of the survey. Patients who had experienced orthopedic implants more than one year earlier were not excluded because 96% of the patients reported improved pain [12]. Finally, we did not exclude patients with chronic pain prior to ICU admission.

Recruitment process

The medical records of patients discharged from the emergency center two to 2.5 years ago were first examined to identify those who met the inclusion criteria. Next, patients were contacted via telephone to confirm that they did not meet the exclusion criteria. A mail survey of eligible patients was then conducted. This consisted of a survey form, a research description, and a consent form. Patients read the description and if they agreed to participate, signed and returned the consent form. Patients who could not be reached after three or more phone calls were considered unreachable and were excluded. If the survey was not returned two weeks after it was sent, the researcher telephoned to follow up. After the initial screening, the same process was conducted every month until the target sample size was achieved.

Data collection

The primary outcome measure was the prevalence of new-onset chronic pain. The secondary outcomes were intensity, location, interference with daily life, and neuropathic pain due to new-onset chronic pain.

We determined the presence of chronic pain, both current and before admission to the emergency center, by asking the survey question, "In the last 24 hours, have you had pain that lasts or recurs for longer than 3 months?" New-onset chronic pain was defined as the presence of current chronic pain and absence of chronic pain before admission to the emergency center.

We measured the intensity, interference with daily life, and location of chronic pain using the Japanese version of the Brief Pain Inventory (BPI). The BPI was developed to measure pain in patients with cancer [13]. This scale has been validated for reliability (Cronbach's alpha > 0.8) and validity (moderately strong relationships of r > 0.5 with Short-Form 36 body pain and Chronic Pain Grade) in patients with arthritis and low back pain [14] and has been translated into Japanese [15]. The BPI is most commonly used to investigate chronic pain in critically ill patients [1]. The Japanese version (BPI-J) comprises factors that include pain intensity, interference with daily life, and location. Pain intensity is an assessment of symptoms within 24 hours and consists of four items: worst, least, average, and right now. The mean of those four items was used as the outcome of chronic pain intensity. Interference with daily life with pain consists of seven aspects: general activity, mood, walking ability, normal work, relations with other people, sleep, and enjoyment of life. This item assesses the extent to which the patient's life is interrupted by pain during 24 hours. We used the mean value of these seven items as the outcome of interference with daily life due to chronic pain. Pain intensity and interference with daily life were measured using a scale of 0-10. A score of "0" indicated no pain and no interference with daily life, while a "10" indicated severe pain and severe interference with daily life. Based on previous studies [16], we defined the outcome of chronic pain intensity and interference with daily life as 1-3 as mild, 4-6 as moderate, and 7-10 as severe. The location of the pain was identified using a body chart in the BPI.

We used the Japanese version of the painDETECT questionnaire (PDQ) to assess neuropathic chronic pain. The PDQ has a sensitivity of 85% and a specificity of 80% [17]. The PDQ has also been used to screen for neuropathic pain in patients with various diseases including osteoarthritis, thoracotomy, and musculoskeletal conditions [18]. It has also been translated into Japanese (PDQ-J) and tested for its reliability and validity [19]. The PDQ is a three-component scale. The first component is a seven-item pathological pain sensation with a scoring range from never (0 points) to very severe (5 points). The score for this component was the sum of the seven items, ranging from 0 to 35. The second component was the course of pain, in which one of the four figures was selected as the most applicable. The third component is radiating pain, which involves spreading the pain to other body parts. This component scored two points for the presence of radiating pain. The PDQ score is the sum of components 1 to 3 and ranges from 0 to 38. In a domestic report of spinal cord injury patients in Japan, the PDQ-J total score was reported to have a sensitivity of 50% and specificity of 92% for a cut-off value of 19 points, a sensitivity of 75%, a specificity of 61% for a cut-off value of 13 points, and a sensitivity of 88% for a cut-off value of 11 [20]. Therefore, we defined a total score of ≥19 as having neuropathic pain (highly likely), a score of 13-19 as possible neuropathic pain, and a score of <13 as no neuropathic pain (unlikely). This cut-off value was similar to that used in previous studies in Japan [21,22].

We retrospectively collected data from the patients’ medical records during hospitalization. Data collected included age, sex, primary diagnosis, Charlson Comorbidity Index, source of admission (out-of-hospital, transfer, or general ward), time between discharge from the emergency center and sending the survey, presence of analgesic use before emergency center admission, history of psychiatric illness, Acute Physiology and Chronic Health Evaluation II (APACHE II) score, Sequential Organ Failure Assessment (SOFA) score, ventilator use, presence of surgery, C-reactive protein >100 mg/L, use of sedative drugs (midazolam, propofol, dexmedetomidine hydrochloride), ICU stay, emergency center stay, and hospital stay. The APACHE II and SOFA scores were calculated using data within 24 h of admission to the ICU or HCU. Based on a previous study [23], C-reactive protein levels >100 mg/L were defined as hyperinflammation. We collected data regarding both non-responders and responders.

Sample size

We estimated the prevalence of chronic pain after critical illness from a previous study to be 35% [2] with a confidence interval of 95% and an acceptable error of 0.12. Calculation of the sample size showed that the required number of patients was 61. Assuming a response rate of 70%, we aimed to mail the questionnaire to 88 patients.

Statistical analysis

Descriptive statistics were calculated. Unless otherwise specified, continuous variables were expressed as medians and interquartile ranges (IQR), and binary variables were expressed as proportions and 95% confidence intervals (95% CI). For comparisons between two groups, Fisher's exact probability test was used for two or more categorical variables and Mann-Whitney’s U test for continuous variables, unless otherwise specified.

We compared the characteristics of responders and non-responders to the survey to assess selection bias.

Our primary outcome was the prevalence of new-onset chronic pain and our secondary outcome was the intensity of chronic pain. Chronic pain intensity was expressed as the mean and standard deviation (SD) and further categorized as mild (1-3), moderate (4-6), or severe (7-10).

Patients with new-onset chronic pain were reported as the number and percentage of patients, along with the location of pain. Interference with daily life was expressed as the mean and standard deviation (SD) and was further categorized as mild (1-3), moderate (4-6), or severe (7-10). The presence of neuropathic pain was described according to the PDQ-J score and classified as neuropathic pain present (highly likely), neuropathic pain possible, and neuropathic pain absent (unlikely).

We analyzed the association between new-onset chronic pain and the intensity of chronic pain with time after discharge from the emergency center. Logistic regression analysis was conducted with new-onset chronic pain as the dependent variable and the time since discharge from the emergency center as the explanatory variable. Similarly, ordinal logistic regression analysis was used, with the intensity of chronic pain as the dependent variable and the duration since discharge from the emergency center as the explanatory variable.

Subgroup analysis was performed to investigate the characteristics of patients with new-onset chronic pain. We divided the descriptive statistics into two groups: with and without new-onset chronic pain. Additionally, patients were divided according to ICU admission status and descriptive statistics were obtained.

We excluded missing items and performed a complete case analysis because we assumed that any missing items were completely random. However, for the BPI pain interference with daily life score, if four or more of the seven items were answered, the mean score of the answered items was imputed [24].

All statistical tests were two-tailed with a statistical significance level of 0.05. Statistical software used was Stata 16.1 (StataCorp, College Station, TX, USA) and R (version 4.1.2, The R Foundation for Statistical Computing Platform, Vienna, Austria).

Ethical considerations

We confirmed that the patients understood the study description and agreed to participate when they signed the consent form and returned it to the participating institutions. Data collection in patients admitted to the emergency center and hospital, including non-respondents, was conducted using an opt-out survey. These methods of informed consent were approved by the Ethics Review Board of Sapporo City University Graduate School of Nursing (11 2021-12-22) and the Hospital Director of Sapporo Medical University Hospital (332-160 2021-12-22).

## Results

**Figure 1:**
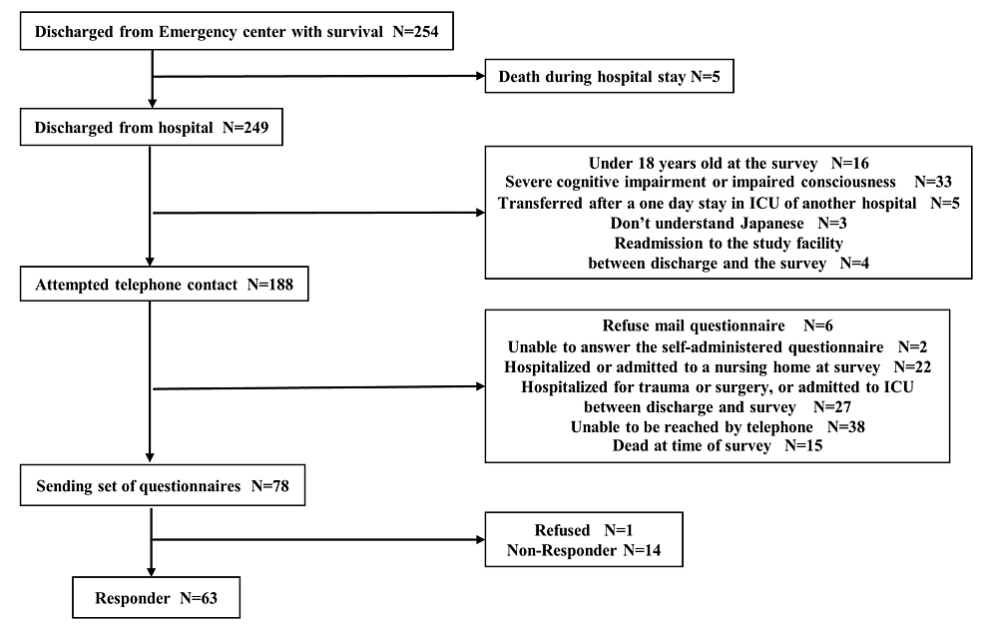
Flowchart of the patient recruitment process.

A flowchart of the enrollment process is shown in Figure 1. The survey questionnaire was mailed to 78 enrolled patients and 64 (82%) responded. Of the 64 patients who returned the survey, one refused to participate in the study. Therefore, data from 63 patients (81%) were analyzed.

One patient (1.6%) was missing an item and also one of the seven BPI-J items related to interference with daily life was missing. Thus, missing items were imputed using the previously mentioned method.

The characteristics of the responders and non-responders to the survey are described in Table 1. The responders were older, with a median age of 59.0 years (IQR 42.5-73.5), and the non-responders had a median age of 36.5 years (IQR 29.3-53.0). There were no statistically significant differences in the APACHE II scores or the proportion of ICU admissions between the two groups. Seventy-nine responders and non-responders had a median of 27 months (IQR 25.0-28.0) since discharge from the emergency center to the time of the survey, with a maximum of 30 months.

**Table 1:** Patient characteristics of responders and non-responders. IQR, interquartile range; APACHE II, Acute Physiology and Chronic Health Evaluation II; SOFA, Sequential Organ Failure Assessment; CCI, Charlson Comorbidity Index; ICU, intensive care unit; LOS, length of stay (days). * Intensive care unit and High-dependency care unit length of stay (days).

Variables	Responders n=63	Non-responders n=14	p-value
Age (years), median (IQR)	59.0 (42.5-73.5)	36.5 (29.3-53.0)	0.022
Female, n (%)	23 (37)	2 (14)	0.12
APACHE Ⅱ, median (IQR)	11.0 (8.0-19.5)	8.0 (5.3-13.8)	0.067
SOFA, median (IQR)	3.0 (1.0-6.0)	2.0 (0.0-4.8)	0.27
CCI, median (IQR)	2.0 (0.0-4.0)	0.5 (0.0-2.8)	0.3
Source of admission, n (%)			
Outside the hospital	41 (65)	12 (86)	0.35
General ward	1 (1.6)	0 (0)	
Another hospital	21 (33)	2 (14)	
Mental illness history, n (%)	7 (11)	2 (14)	0.66
Analgesic use before admission, n (%)	14 (22)	3 (21)	1.0
Number of months to send the survey, median (IQR)	27.0 (25.0-28.5)	25.0 (25.0-26.8)	0.075
Primary diagnosis at admission, n (%)			
Trauma	29 (46)	5 (36)	0.62
Cardiogenic	9 (14)	1 (7.1)	
Central nervous system	4 (6.3)	0 (0.0)	
Hemorrhagic shock	2 (3.2)	1 (7.1)	
Respiratory failure	2 (3.2)	0 (0.0)	
Toxicosis	6 (9.5)	3 (21)	
Infection	5 (7.9)	1 (7.1)	
Others	6 (9.5)	3 (21)	
Ventilator use, n (%)	16 (25)	2 (14)	0.49
Surgery, n (%)	29 (46)	3 (21)	0.14
Sedation use, n (%)	16 (25)	2 (14)	0.49
ICU admission, n (%)	39 (62)	8 (57)	0.77
ICU LOS, median (IQR)	0.6 (0.0-2.2)	0.8 (0.0-2.1)	0.88
Emergency center LOS*, median (IQR)	7.0 (2.7-15.3)	7.1 (0.7-13.9)	0.43
Hospital LOS, median (IQR)	15.0 (5.3-27.6)	7.1 (0.7-18.5)	0.032

Prevalence and intensity of chronic pain

Of the 63 patients, 12 had chronic pain, with a prevalence of 19% (95% CI:10.2-30.9). Also, three (4.8%) patients had chronic pain during the survey and before admission to the emergency center. The prevalence of new-onset chronic pain was 14% (95% CI:6.7-25.4).

The mean intensity of chronic pain among the new-onset patients was 3.8 (SD 1.1). Six of the nine patients (67%) had moderate or severe symptoms (Table 2).

**Table 2:** Pain intensity and interference with daily life in patients with new-onset chronic pain after discharge from the tertiary emergency center. BPI, Japanese version of the Brief Pain Inventory; SD, standard deviation.

Variables	n=9
BPI pain intensity score, mean (SD)	3.8 (1.1)
BPI pain intensity category, n (%)	
None (0)	0 (0.0)
Mild (1-3)	3 (33)
Moderate (4-6)	6 (67)
Severe (7-10)	0 (0.0)
BPI pain interference score, mean (SD)	4.2 (2.4)
BPI pain interference category, n (%)	
None (0)	0 (0.0)
Mild (1-3)	3 (33)
Moderate (4-6)	5 (56)
Severe (7-10)	1 (11)

Location of chronic pain

**Figure 2:**
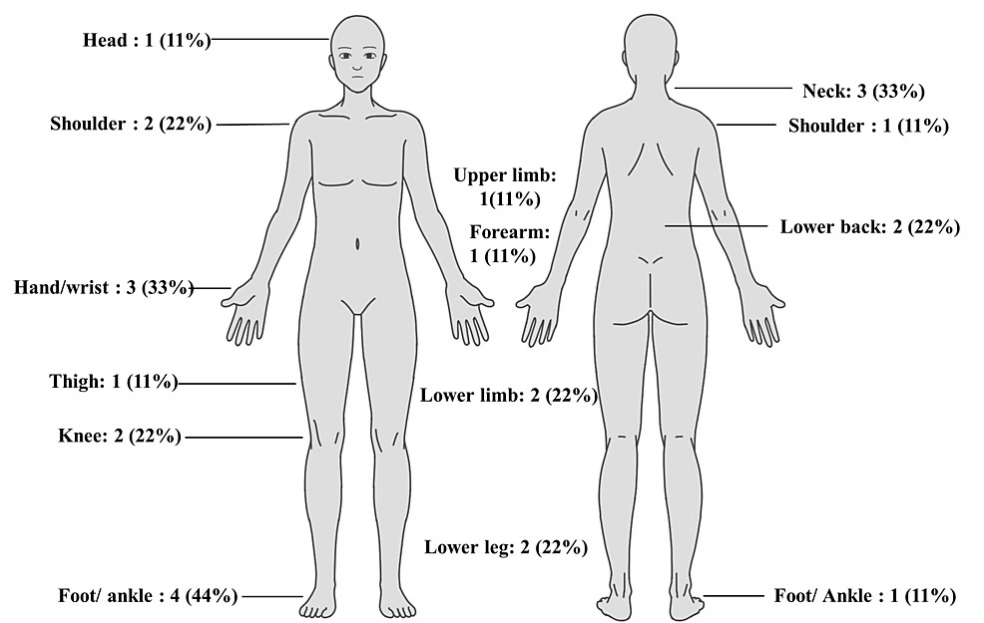
Location of new onset chronic pain (N=9)

The location of the pain reported by the nine patients with new-onset chronic pain is shown in Figure 2. The most frequently reported location of chronic pain was the anterior of the foot/ankle, with four (44%) patients reporting pain, followed by the anterior of hand/wrist and posterior neck, with three (33%) patients each.

Interference with daily life by chronic pain

The mean score for interference with daily life in the nine patients with new-onset chronic pain was 4.2 (SD 2.4). Six of the nine patients (67%) had moderate or severe symptoms (Table 2).

Presence of neuropathic pain

Of the nine patients with new-onset chronic pain, four (44%) had neuropathic pain on the PDQ-J, zero (0%) had possible neuropathic pain, and five (56%) had no neuropathic pain.

Presence of chronic pain and the time since discharge

There was no significant association (OR=1.16; 95% CI=0.80-1.68; P=0.44) between new-onset chronic pain and the time since discharge.

Intensity of chronic pain and the time since discharge

**Figure 3:**
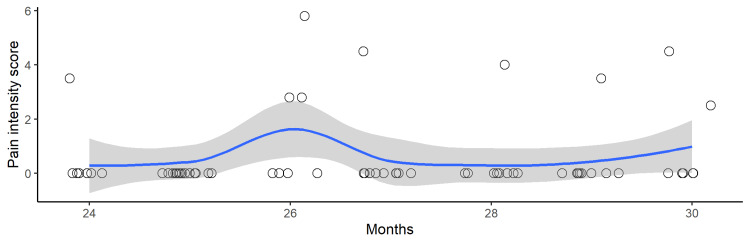
Scatterplot of BPI pain intensity score of new-onset chronic pain vs. months since discharge from the emergency center (N=63). The vertical axis represents the intensity of new-onset chronic pain as measured by the BPI, and the horizontal axis represents the number of months since discharge. The blue line represents the Loess curve, and the gray line represents the 95% confidence interval. BPI, Japanese version of the Brief Pain Inventory

The relationship between the intensity of new-onset chronic pain and time since discharge from the emergency center is shown in Figure 3. There was no significant association (OR=1.15; 95% CI=0.80-1.66; p=0.45) between chronic pain intensity and time since discharge.

Subgroup analysis

Table 3 shows the characteristics of the groups with and without new-onset chronic pain. The group with new-onset chronic pain had more patients who underwent surgery and had longer emergency center and hospital stays. There were no statistically significant differences in the APACHE II scores, ventilator use, or ICU admission.

**Table 3:** Subgroup analysis between with and without new-onset chronic pain. IQR, interquartile range; APACHE II, Acute Physiology and Chronic Health Evaluation II; SOFA, Sequential Organ Failure Assessment; CCI, Charlson Comorbidity Index; ICU, intensive care unit; LOS, length of stay (days). * Number of days with C-reactive protein level > 100 mg/L. † Intensive care unit and High-dependency care unit length of stay (days).

Variables	Without New-onset chronic pain n=54	With New-onset chronic pain n=9	p-value
Age (years), median (IQR)	66.5 (45.3-75.5)	47.0 (42.0-51.0)	0.14
Female, n (%)	34 (63)	6 (67)	1.0
APACHE Ⅱ, median (IQR)	11.0 (8.0-19.8)	8.0 (6.0-13.0)	0.1
SOFA, median (IQR)	3.0 (1.0-6.0)	3.0 (2.0-7.0)	0.82
CCI, median (IQR)	2.0 (0.0-4.0)	1.0 (0.0-1.0)	0.058
Source of admission, n (%)			
Outside the hospital	35 (65)	6 (67)	1.0
General ward	1 (1.9)	0 (0.0)	
Another hospital	18 (33)	3 (33)	
Presence of chronic pain before admission, n (%)	7 (13)	0 (0.0)	0.58
Mental illness history, n (%)	7 (13)	0 (0.0)	0.58
Analgesic used before admission, n (%)	13 (24)	1 (11)	0.67
Number of months to send the survey, median (IQR)	27.0 (25.0-28.0)	27.0 (26.0-29.0)	0.43
Primary diagnosis at admission, n (%)			
Trauma	22 (41)	7 (78)	0.72
Cardiogenic	8 (15)	1 (11)	
Central nervous system	3 (5.6)	1 (11)	
Hemorrhagic shock	2 (3.7)	0 (0.0)	
Respiratory failure	2 (3.7)	0 (0.0)	
Toxicosis	6 (11)	0 (0.0)	
Infection	5 (9.3)	0 (0.0)	
Others	6 (11)	0 (0.0)	
Hyperinflammationdays*, median (IQR)	0.0 (0.0-1.0)	2.0 (0.0-4.0)	0.18
Ventilator use, n (%)	14 (26)	2 (22)	1.0
Surgery, n (%)	21 (39)	8 (89)	0.009
Sedation use, n (%)	14 (26)	2 (22)	1.0
ICU admission, n (%)	33 (61)	6 (67)	1.0
ICU LOS, median (IQR)	0.6 (0.0-2.1)	0.8 (0.0-2.2)	0.69
Emergency center LOS†, median (IQR)	6.0 (2.0-11.8)	24.5 (18.5-26.3)	<0.001
Hospital LOS, median (IQR)	12.3 (4.1-23.0)	26.3 (24.5-64.1)	0.003

Table 4 shows the characteristics of the ICU and non-ICU groups. The median APACHE II score was 15.0 (IQR 9.0-24.5) in the ICU group and 8.0 (IQR 5.8-10.3) in the non-ICU group, and this difference was significant (p<0.001). There were no statistically significant differences in the presence of new-onset chronic pain, the categories of intensity and interference with daily life from new-onset chronic pain.

**Table 4:** Subgroup analysis between non-ICU admission and ICU admission. IQR, interquartile range; APACHE II, Acute Physiology and Chronic Health Evaluation II; SOFA, Sequential Organ Failure Assessment; CCI, Charlson Comorbidity Index; ICU, intensive care unit; LOS, length of stay (days): BPI, Japanese version of the Brief Pain Inventory. * Admission to high-dependency care unit only. † Number of days with C-reactive protein level > 100 mg/L. ‡ Intensive care unit and High-dependency care unit length of stay (days)

Variables	Non-ICU admission* n=24	ICU admission n=39	p-value
Age (years), median (IQR)	56.0 (39.0-71.5)	62.0 (45.5-73.5)	0.54
Female, n (%)	10 (42)	13 (33)	0.59
APACHE Ⅱ, median (IQR)	8.0 (5.8-10.3)	15.0 (9.0-24.5)	<0.001
SOFA, median (IQR)	1.0 (0.0-2.3)	5.0 (2.5-7.5)	<0.001
CCI, median (IQR)	1.0 (0.0-3.0)	2.0 (1.0-4.0)	0.22
Source of admission, n (%)			
Outside the hospital	12 (50)	29 (74)	0.07
General ward	1 (4.2)	0 (0.0)	
Another hospital	11 (46)	10 (26)	
Presence of chronic pain before admission, n (%)	2 (8.3)	5 (13)	0.69
Mental illness history, n (%)	0 (0.0)	7 (18)	0.038
Analgesic used before admission, n (%)	3 (13)	11 (28)	0.22
Number of months to send the survey, median (IQR)	26.5 (25.0-28.0)	27.0 (25.0-29.0)	0.35
Primary diagnosis at admission, n (%)			
Trauma	13 (54)	16 (41)	0.19
Cardiogenic	2 (8.3)	7 (18)	
Central nervous system	1 (4.2)	3 (7.7)	
Hemorrhagic shock	0 (0.0)	2 (5.1)	
Respiratory failure	0 (0.0)	2 (5.1)	
Toxicosis	1 (4.2)	5 (13)	
Infection	2 (8.3)	3 (7.7)	
Others	5 (21)	1 (2.6)	
Hyperinflammation days†, median (IQR)	0.0 (0.0-0.0)	1.0 (0.0-3.0)	<0.001
Ventilator use, n (%)	0 (0.0)	16 (41)	<0.001
Surgery, n (%)	10 (42)	19 (49)	0.61
Sedation use, n (%)	0 (0.0)	16 (41)	<0.001
ICU LOS, median (IQR)	0.0 (0.0-0.0)	1.3 (0.8-4.0)	<0.001
Emergency center LOS‡, median (IQR)	4.1 (1.1-12.3)	9.0 (3.8-16.9)	0.12
Hospital LOS, median (IQR)	10.7 (1.2-21.6)	18.7 (9.0-36.2)	0.03
Presence of new-onset chronic pain, n (%)	3 (13)	6 (15)	1.0
BPI pain intensity category, n (%)			
None (0)	21 (88)	33 (85)	0.334
Mild (1-3)	2 (8.3)	1 (2.6)	
Moderate (4-6)	1 (4.2)	5 (13)	
Severe (7-10)	0 (0.0)	0 (0.0)	
BPI pain interference category, n (%)			
None (0)	21 (88)	33 (85)	1.0
Mild (1-3)	1 (4.2)	2 (5.1)	
Moderate (4-6)	2 (8.3)	3 (7.7)	
Severe (7-10)	0 (0.0)	1 (2.6)	

## Discussion

We investigated the prevalence, location, and interference with daily life of patients with chronic pain discharged from a single-center tertiary emergency center using questionnaires and medical records. The prevalence of chronic pain was 19%, and new-onset chronic pain was 14%. In addition, approximately 70% of patients with new-onset chronic pain had more than moderate intensity and degree of interference with daily life. The most frequent location of new-onset chronic pain was the foot/ankle, followed by the hand/wrist and posterior neck; nearly half of the patients had neuropathic pain.

The prevalence of chronic pain was lower than that reported previously [1]. There are several possible reasons for this. First, differences in the definition of measured pain may have affected the difference in prevalence from previous studies. Indeed, each prior study has indicated differences in the definition of pain after critical illness [1]. For example, investigating two years after ICU discharge, one study defined chronic pain as "graded chronic pain scale >0 (a score indicating at least low-intensity pain and disability)" [25] and another study defined pain as "BPI pain intensity >4" [4]. We used the IASP definition of chronic pain as the duration or recurrence of pain [9], regardless of the intensity of the pain measured. Previous studies may have reported more chronic pain because of these differences in definitions.

Second, the length of time between discharge from the emergency center and the timing of the survey may have influenced the differences in prevalence between this and previous studies. Many previous studies have reported up to one year after critical illness, and few have investigated chronic pain in patients more than two years later. One study reported that one year after discharge from the ICU, 45% of patients who reported new-onset (or worsening) chronic pain after ICU admission improved 27-44 months after discharge from the ICU [23]. A longitudinal study that followed sepsis and septic shock patients for two years after leaving the ICU reported a prevalence of chronic pain of 61% at six months, 76% at one year, and 83% at two years since ICU discharge [25]. The first study followed only patients with chronic pain [23] and the second followed all study cohorts [25]. Therefore, it is difficult to determine how the time between critical illness and the timing of the survey affects prevalence. Nevertheless, we believe that this may have affected the difference in prevalence from prior studies.

Third, differences in the exclusion criteria may have affected the prevalence. Previous studies did not consider trauma, surgery, or ICU admission events that occurred between discharge and the timing of the survey [2,4,5,25]. Such events occurring during the follow-up period could have affected the measurement of pain in the survey. To accurately measure chronic pain due to critical illness, we excluded patients who experienced these events.

We believe that the severity of the patients’ illness in our study did not contribute to the difference in prevalence from prior studies. The effect of severity on pain after a critical illness has been reported in previous studies. One study indicated that the patient's severity score may be a risk factor for pain one year after ICU discharge, although it did not find statistical significance [2]. However, other studies also have found no association between patient severity scores and chronic pain after ICU discharge [3,23]. Most previous studies included only patients admitted to the ICU, and many studies included patients with relatively high-severity illnesses among those admitted to the ICU. For example, one study included patients who stayed in the ICU for >48 hours with a mean APACHE II score of 16.0 [8] whilst another study included patients with severe sepsis and septic shock with a mean APACHE II score of 16.9 [26]. Since our study also included patients who were only admitted to the HCU, the impact of the difference in severity from previous studies needs to be considered. However, also based on the results of our subgroup analysis, we believe that it is unlikely that the severity of the illness affected the differences in the prevalence of chronic pain between our study and previous studies.

Patients with new-onset chronic pain two to 2.5 years since discharge may have more severe pain intensity and degree of interference with daily life than those with pain one year after critical illness. For example, in a previous study of ICU patients with respiratory failure, septic shock, and cardiogenic shock, of 187 patients with pain one year after discharge from the ICU, 89 (48%) had moderate pain intensity (pain intensity score ≥ 5 on the BPI) or greater, and a moderate degree of interference with daily life (interference with daily life score ≥ 5 on the BPI) or greater was reported for 56 (30%) [5]. That study had a smaller proportion of patients with moderate to severe symptoms than our study, even though the time from severe illness to the investigation was shorter and there was no duration or recurrence condition for the pain being measured. However, the definition of moderate disease in the classification of pain intensity and interference with daily life scores differs from that in our study (BPI-J score of ≥4 was moderate in our study); therefore, caution should be exercised in interpretation. In addition, the proportion of patients with neuropathic pain may have influenced the higher number of patients in our study with new-onset chronic pain, who had worse pain intensity and interference with daily life. Previous studies have reported that, among patients with chronic pain, those with neuropathic pain have worse pain intensity and degree of interference with daily life [27]. Thus, we believe that neuropathic pain also affects pain intensity and interferes with daily life.

Our study had several strengths. First, we used the latest definition of chronic pain and validated tools for measuring pain characteristics. Second, we measured the prevalence and characteristics of chronic pain in long-term survivors two or more years after discharge from a tertiary emergency center. Studies on chronic pain in long-term survivors after a critical illness are rare. Third, the patients included were emergency patients, not only those admitted to the ICU, but also critically ill patients admitted to the HCU. Fourth, to the best of our knowledge, this is the first Asian study to investigate chronic pain in patients after critical illness.

Our study also had several limitations. First, 38 patients (15% of those screened) could not be traced because they could not be reached by phone. We excluded these patients but could not determine if they had died, were in distress, or were unable to complete the survey; we presume that the main reasons were death, hospitalization or admission to a nursing home, relocation, or busyness rather than pain-related reasons. Therefore, this may have had little impact on the results. Second, recall bias was possible because the survey asked about the presence of chronic pain before admission to the emergency center. However, few patients (only three patients, 4.8% of responders) experienced chronic pain both at the time of the survey and before admission to the emergency center. Thus, we believe that the actual prevalence of new-onset chronic pain would be lower, even if bias was present. Third, the sample size was limited because the facility in which this study was conducted had changed its ward structure owing to the SARS-CoV-2 epidemic, which resulted in a ward dedicated to SARS-CoV-2 patients. However, the patients in our study were diverse, with 46% undergoing surgery and 62% admitted to the ICU. Fourth, due to the long period of time between discharge and survey completion, new-onset chronic pain may not have been associated with admission to the emergency center. Fifth, our study was a single-center study, and the patients were admitted to the tertiary emergency center according to Japanese domestic standards. Moreover, some patients were excluded because they were unable to complete the self-administered questionnaire (e.g., due to dementia) or communicate via telephone. For these reasons, external validity needs to be considered, and further investigation is warranted.

Further extensive longitudinal studies with uniform definitions of chronic pain in long-term survivors after critical illness are needed. Provision of information and post-discharge follow-up to patients after critical illness is required because even long-term survivors may experience chronic pain.

## Conclusions

According to the new IASP definition of chronic pain, we found a lower prevalence of new-onset chronic pain among long-term survivors after discharge from a tertiary emergency center than previous studies. However, many patients with new-onset chronic pain experience more than moderate pain intensity and interference with daily life. This study represents the first-step towards understanding and adequately managing chronic pain after critical illness.
